# Can plasmonic Al nanoparticles improve absorption in triple junction solar cells?

**DOI:** 10.1038/srep11852

**Published:** 2015-07-03

**Authors:** L. Yang, S. Pillai, M. A. Green

**Affiliations:** 1 Australian Centre for Advanced Photovoltaics, University of New South Wales, Sydney, NSW-2052, Australia; 2 College of Applied Nuclear Technology and Automation Engineering, Chengdu University of Technology, Chengdu 610059, China

## Abstract

Plasmonic nanoparticles located on the illuminated surface of a solar cell can perform the function of an antireflection layer, as well as a scattering layer, facilitating light-trapping. Al nanoparticles have recently been proposed to aid photocurrent enhancements in GaAs photodiodes in the wavelength region of 400–900 nm by mitigating any parasitic absorption losses. Because this spectral region corresponds to the top and middle sub-cell of a typical GaInP/GaInAs/Ge triple junction solar cell, in this work, we investigated the potential of similar periodic Al nanoparticles placed on top of a thin SiO_2_ spacer layer that can also serve as an antireflection coating at larger thicknesses. The particle period, diameter and the thickness of the oxide layers were optimised for the sub-cells using simulations to achieve the lowest reflection and maximum external quantum efficiencies. Our results highlight the importance of proper reference comparison, and unlike previously published results, raise doubts regarding the effectiveness of Al plasmonic nanoparticles as a suitable front-side scattering medium for broadband efficiency enhancements when compared to standard single-layer antireflection coatings. However, by embedding the nanoparticles within the dielectric layer, they have the potential to perform better than an antireflection layer and provide enhanced response from both the sub-cells.

It has been demonstrated that multiple and high-angle light scattering from metallic plasmonic nanoparticles (NPs)^1^,^2^,^3^,^4^ can improve light absorption in solar cells and related devices through nanoscale light trapping. Parasitic absorption of metallic NPs and interference losses at the wavelengths below resonance frequency^5^ can reduce the effectiveness of the NPs when they are located on the front of Si and GaAs solar cells. This is particularly evident for silver and gold nanoparticles, where high-index substrates such as Si can red-shift the resonance wavelengths further into the visible spectrum, thereby impeding useful absorption of the high-energy wavelength spectrum. Rear-located metal NPs and dielectric nanostructures were then investigated to circumvent this problem^6^,^7^,^8^. Recently, Al with a plasma frequency in the ultraviolet has attracted attention due to its lower parasitic absorption over the solar spectrum and its ability to scatter light in the entire visible region^7^,^8^,^9^,^10^,^11^. Although front-located Al NPs were not considered beneficial for crystalline silicon solar cells due to their strong absorption band at approximately 800 nm^12^, recent studies have shown front-located Al NPs to be advantageous^13^,^14^. The motivation for this work was the results reported recently in which a 22% integrated efficiency enhancement was obtained by locating Al NPs on the top surface of GaAs photodiodes^11^. The spectral region of 400 nm–900 nm, where efficiency enhancements were reported, is of interest to triple junction solar cells (3JSC), which have a similar response region for top GaInP and middle GaInAs sub-cells. Hence, we investigated the potential of Al nanoparticles for improving the efficiency of 3JSC.

The high-efficiency of multi-junction solar cells has been particularly attractive for cost-effective terrestrial concentrator systems^15^. Interest was stimulated when a milestone record efficiency of 40.7% was achieved via an upright metamorphic 3-junction GaInP/GaInAs/Ge concentrator cell^16^,^17^. III–V multi-junction concentrated photovoltaics (CPV technology continues to grow rapidly in efficiency.

The state-of-the-art commercially available 3JSC in a CPV system is a monolithically stacked Ga_0.50_In_0.5_0P/Ga_0.99_In_0.01_As/Ge junction, which has reached conversion efficiencies of 41.6%^18^,^19^,^20^. Theoretical calculations show that the ideal 3JSC device should have respective bandgaps of 1.7 eV and 1.1 eV for the top and middle junctions to achieve current-matching to Ge and maximise efficiency. The bandgap for the upper two junctions is 1.9-1.8 eV and 1.4 eV in the state-of-the-art Ga_0.50_In_0.50_P/Ga_0.99_In_0.01_As/Ge solar cell, which is higher than the ideal bandgap, thus resulting in less current in both sub-cells and leading to a current imbalance between sub-cells^15^.

Because the mismatch results from inefficient light absorption and conversion in the two upper sub-cells, improving the light absorption of these sub-cells is an effective path to solving this problem. Reducing surface reflection in the wavelengths of interest or lowering the bandgap of the upper two junctions via increasing the indium content in alloy III-V material are useful methods that have been adopted^15^,^21^. Conventional AR coatings for 3JSC are composed of a stack of dielectrics with different refractive indices such as MgF_2_/ZnS^22^, Al_2_O_3_/TiO_2_^20^, and MgF_2_/TiO_2_^17^. The best simulated double-layer AR (DLAR) coating of MgF_2_/ZnS showed a 1.6% weighted reflectance over the response spectra (300–650 nm) of the top sub-cell^21^.

The epitaxial growth process for multi-junction cells requires planar geometries, thus making it challenging to apply any sort of traditional texturing or nanotexturing. This fact makes light trapping with optically coupled structures, such as plasmonics, of interest. For III-V multi-junction solar cells, the front configuration for metal NPs would be preferable to the rear one because the Ge bottom cell does not require any additional light trapping. The front-deposited Al NPs could therefore provide a pathway to improving the light absorption of upper sub-cells in 3JSC to alleviate the current mismatch.

Motivated by the recent report of improvement in GaAs photodiodes^11^, we investigated the feasibility of front-deposited Al NPs to improve the external quantum efficiency (EQE) of the top and middle sub-cells in the 3JSC configuration of GaInP/GaInAs/Ge. We compared our results with earlier work^11^ and benchmarked the performance to the case with standard AR coatings. While random metal nanoparticles may have the potential to provide the necessary broadband response to enhance the performance of the top and middle cells combined, uniform arrays are investigated through simulations to aid better control over current matching issue in 3JSCs. In this work we make use of well accepted physical models to study and understand the potential of plasmonics for a 3JSC which has not been previously reported in the literature.

## 3JSC Structure and Simulation

Because the current of a multi-junction solar cell can be limited by either the top or middle cell, it is critical that the light coupling process be as broadband as possible. For this purpose, we benchmark the performance of the two upper sub-cells based on the front reflectivity and the calculated EQE after incorporation of the Al NPs. Ideally, the reflection for each of the sub-cells should be low and the EQE high.

The finite-difference time-domain (FDTD) simulator Lumerical (Lumerical Solutions Inc.) was used to optically model the device. The wavelength-dependent surface reflectance (WDR) and light absorption (WDA) in each sub-cell was directly simulated. Both WDR and WDA are the impulse response of the system excited by a FDTD source of a Gaussian pulse. The solar weighted reflection (SWR) was calculated as^23^.


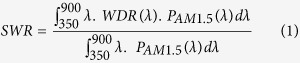


where P_AM1.5_ is the solar spectral power density, which represents the solar spectral irradiance under AM1.5 global radiation.

The internal and external quantum efficiencies (IQE and EQE) are also normally used to characterise the performance of solar cells, defined as the ratio of the number of collected charges to absorbed and incident photons, respectively. Assuming an IQE of 100% (i.e., the generated carriers are fully collected without recombination), the EQE will attain its maximum value and can be calculated solely from an optical simulation of the device. In this study, the calculated EQE is the same as WDA. The EQE was solar weighted and integrated (expressed as SWQE) over wavelengths by using the same equation as for SWR but with the integration over the 350-650 nm range for the top cell and over the 650–900 nm for the middle cell. The short circuit current density for each cell is given by


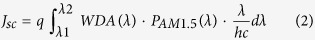


where *h* is Plank’s constant, *c* is the speed of light in free space and *q* is the charge on an electron, with the *λ*_*1*_ and *λ*_*2*_ ranges selected in the same way as for SWQE.

**Figure 1:**
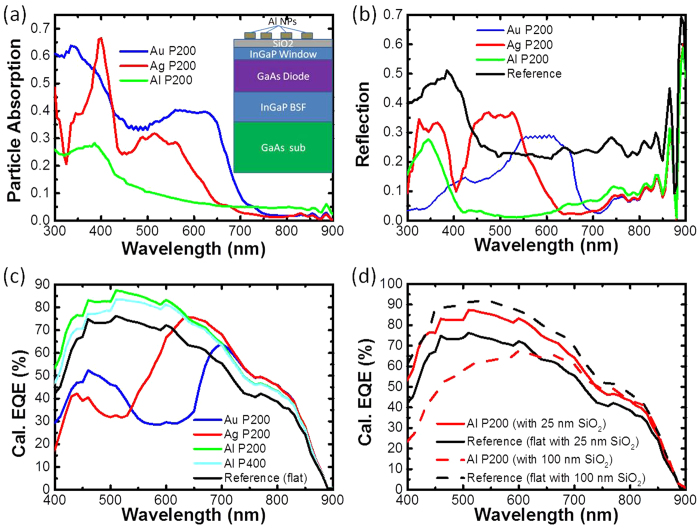
Reproduced calculations for a GaAs cell for the photodiode structure reported in Ref 11 for a 100 nm diameter and 50 nm height Al nanoparticle array for different pitch as indicated : (**a**) Absorption in NPs (inset: device structure), (**b**) surface reflection, (**c**) calculated EQE and (**d**) calculated EQE for a thicker SiO_2_ layer.

As a check, we first replicate the calculation for single-junction GaAs photodiodes as reported by others^11^ and find our results comparable with those published. The GaAs cell comprises a 25-nm SiO_2_ spacer layer, a 30-nm InGaP window layer, a 500-nm InGaP back surface field layer and a 500-nm active GaAs region on a GaAs substrate (see Fig. 1a inset). The nanoparticle diameter, height and period are 100, 50 and 200 nm, respectively with an additional period of 400 nm for the Al NP. As shown in Fig. 1, the NP absorption spectra (Fig. 1(a)) and the device reflection spectra (Fig. 1(b)) in our calculations are very similar to those reported by other workers^11^ at most wavelengths. Because 100% IQE is considered in these calculations, the simulated results represent the upper limit EQE of the device. This explains why our calculated EQE in Fig. 1(c) yielded similar trends but with larger values than those from the literature because these were experimental EQE results. In Fig. 1(d), the reference cell and the cell with Al NPs for the 25-nm SiO_2_ thickness from the original study are compared to the case of the 100 nm-thick SiO_2_ AR coating with and without the Al NPs and will be discussed more in the later section. The reference cell reported in the literature^11^ is a cell without NPs but with the same thickness of the SiO_2_ layer (25 nm) as with the NPs.

As the results in Fig. 1 show, Al NPs gave promising results, with the Al interband transition at approximately 800 nm having no detrimental effect on the photocurrent compared to the reference cell. The spectral range from 400–900 nm was improved, which is the same spectral range for the two top sub-cells in the 3JSC structure. We hence used these results to further extend our scope to 3JSC to address light-trapping problems and investigate the potential for any improvement.

**Figure 2:**
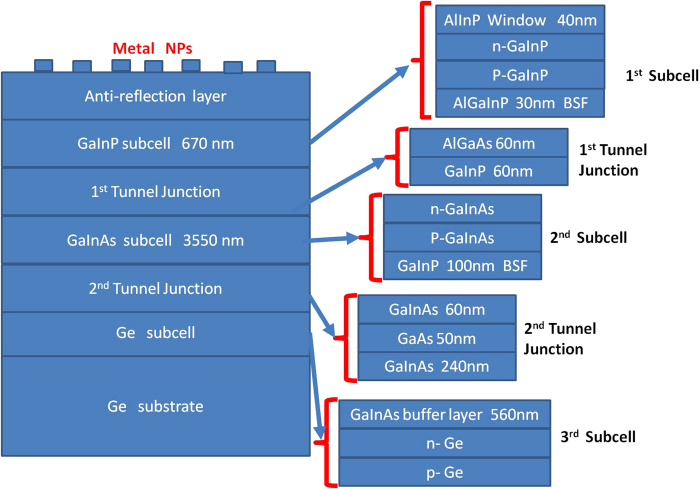
Schematic of GaInP/GaInAs/Ge solar cell device structure used in the simulation.

It is well known that a typical monolithic 3JSC device has three stacks of cells with different bandgaps, which are connected by wide bandgap tunnel junctions^18^,^24^,^25^. The nearly 20 layers in a 3JSC are normally fabricated by molecular organic chemical vapour deposition (MOCVD)^27^. Although the GaInP/GaInAs/Ge 3JSC has been commercialised and already applied to CPV, the structural details, including specific layer thickness, material component and doping parameters, are not in the public domain. Hence, a device structure based on published literature^25^,^26^,^27^,^28^ is chosen as our simulated structure of GaInP/GaInAs/Ge 3JSC, which is shown in Fig. 2. The ternary alloy Ga_0.51_In_0.49_P was used as the back surface field layer for the middle sub-cell, the first tunnel junction, and the active material of the top sub-cell. Another main ternary alloy, Ga_0.99_In_0.01_As, was selected as the buffer layer, the second tunnel junction and the p-n junction of the middle sub-cell. Periodic cylindrical Al NPs of 50 nm height were placed on the front top surface of a SiO_2_ spacer layer. The height of the particles was fixed, with the period, the diameter and the thickness of the underlying SiO_2_ layer taken as variable parameters.

In our 3JSC simulation, the optical constants of GaInAs, GaAs, Ge, SiO_2_, Al, MgF_2_ and ZnS are directly or linearly interpolated from Palik^29^. The optical constants for the quaternary alloy AlGaInP and ternary alloys GaInP, AlGaAs and AlInP are all indirectly derived from the SOPRA *n & k* database.

### Results and Analysis

SWR and SWQE data are used to benchmark the performance of the two sub-cells compared to the reference without NPs. The response of the top and middle sub-cells are investigated independently (SWQE) and together (SWR). The typical response of a GaInP top cell is from 300–650 nm and of a GaInAs middle sub-cell is from 650–900 nm.

### Performance of flat 3JSC with SLAR of SiO_2_ as a reference sample

**Figure 3:**
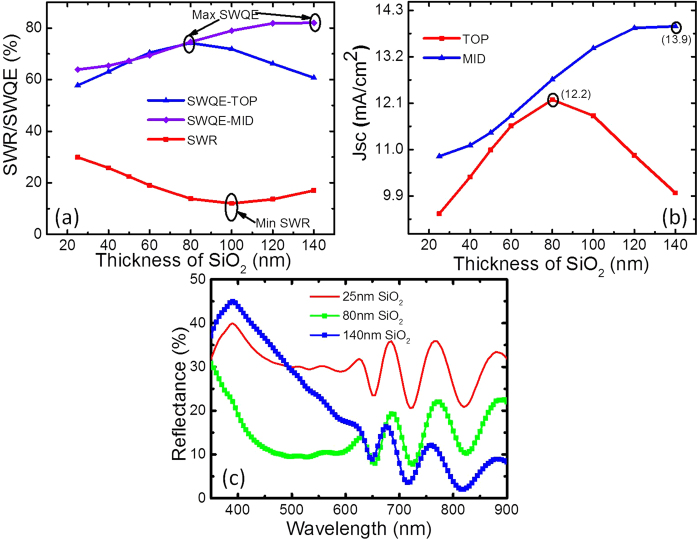
Calculated (**a**) SWR and SWQE (**b**) Short current density for the top and middle sub-cells as a function of the thickness of the SiO_2_ layer without nanoparticles(**c**) Reflection for the optimised SLAR thicknesses of 80 nm and 140 nm for the top and middle sub-cells, respectively, along with the 25 nm spacer layer for comparison.

We calculated the SWR, SWQE for the two upper sub-cells with different thicknesses of SiO_2_ without NPs, with the SiO_2_ acting as a single-layer antireflection coating (SLAR) in this case. As shown in Fig. 3(a), the maxima for SWQE for the top (74.2%) and middle (82%) cells are at 80 nm and 140 nm-thick SLAR, respectively. The minimum SWR is at the thickness of 100 nm, with a value of 12%. The minimum SWR, maximum SWQE for the top cell and middle cell are not at the same thicknesses of SLAR. Fig. 3(a) also shows that the SWQE of the middle cell is higher than that of the top cell for all our calculated SLAR thicknesses, while Fig. 3(b) displays the same trend in the corresponding current density (integration spectrum 350–650 nm for the top cell and 650–900 nm for the middle cell). Therefore, the overall current of the whole device is mainly limited by the top sub-cell. The maximum currents of the top and middle sub-cells were 12.2 and 13.9 mA/cm^2^, respectively. While these values are more applicable to the material, structure and thickness of the layers assumed in this study and might not be a true representation of a typical 3JSC device performance, they do give an indication of the change in response from the two sub-cells. Figure 3(c) shows the reflection for the two different thicknesses of SLAR. Also included is the 25-nm SiO_2_ case for comparison (without NP), which clearly shows significant loss due to reflection. Based on the results from our simulations, 80-nm SiO_2_ gave good overall current and is a good compromise between SWQE and SWR for the two sub-cells. Therefore, it is taken as our reference for comparison purposes and will be referred to as SLAR henceforth.

### Performance of Al-incorporated 3JSC

#### SWR over 350 to 900 nm

**Figure 4:**
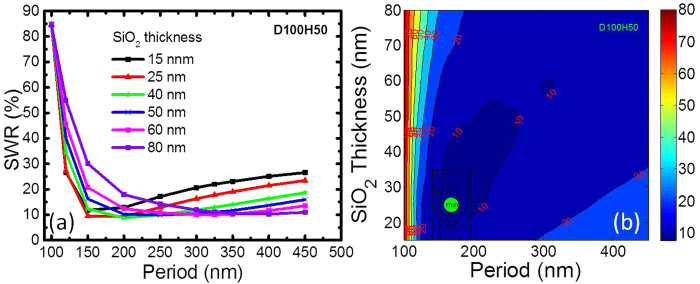
(**a**) SWR as a function of the underlying SiO2 thickness and NP period for D100H50, (**b**) Contour map plot of (**a**) with the green spot for minimum SWR at P168AR25.

Because Al NPs with a diameter of 100 nm and a height of 50 nm demonstrated EQE enhancements from a GaAs cell in Ref 11, we chose the same parameters for our initial simulations. This series of data was labelled D100H50 (D- diameter, H- height, and the related dimensions signify the number in nm) for convenience. The period of the NP array and thickness of the underlying SiO_2_ layer were taken as variable parameters. The period was varied from 100 to 450 nm, and the thickness of the underlying SiO_2_ layer varied from 15 to 80 nm for each period. Figure 4(a) shows the calculated SWR for the D100H50 series. Figure 4(b) shows the interpolated SWR contour map, which is obtained by triangulation-based cubic interpolation of the discrete data in Fig. 4(a). The SWR tends to decrease rapidly with the increase in period up to 200 nm. This finding signifies that denser Al NPs perform more like a highly reflective coating, resulting in the loss of useful light. The calculated minimum SWR is 8.7% for NPs with a period of 200 nm and a SiO_2_ thickness of 40 nm (naming convention used: P200AR40, P- for period of the NPs, AR- for the thickness of the underlying SiO_2_ layer), while the interpolated minimum SWR is 7.5% for NPs with a period of 168 nm and a SiO_2_ thickness of 25 nm.

#### SWQE for top sub-cell over 350 to 650 nm

**Figure 5:**
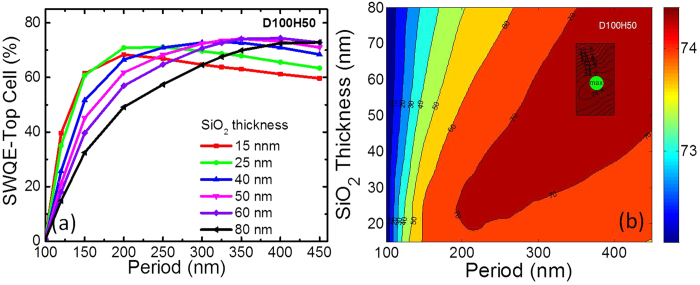
(**a**) SWQE of the top sub-cell as a function of the underlying SiO_2_ thickness and NP period for D100H50, (**b**) Contour map plot. (Green spot indicates the maximum SWQE).

Here, we investigate the response of the GaInP top sub-cell in the spectral region of 350–650 nm. Figure 5(a,b) shows the calculated and interpolated SWQE for the GaInP top sub-cell, respectively. The interpolation method is same as that used in Fig. 4(b). The maximum calculated and interpolated SWQE for the top GaInP sub-cell is 74.4% and 74.5% with NP configurations of P400AR60 and P375AR59, respectively.

#### SWQE for the middle sub-cell over 650 to 900 nm

**Figure 6:**
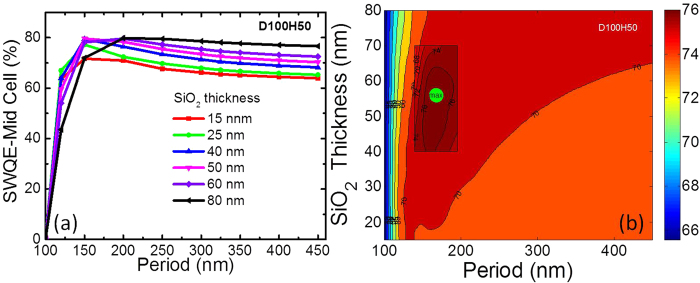
(**a**) SWQE of the middle sub-cell as a function of the underlying SiO_2_ thickness and NP period for D100H50, (**b**) Contour map plot. (Green spot indicates the maximum SWQE).

As shown previously, similar plots were extracted for the middle sub-cell. As shown in Fig. 6(a), the maximum SWQE for the middle cell is located at P200AR80, with a value of 79.8%, while the contour map (see Fig. 6(b)) showed that the maximum is at P168AR56, with a SWQE of approximately 81.6%.

Comparing Figs 4(b), 5(b) and 6(b), it was found that the minimum SWR, maximum SWQE for the top and middle cells did not accurately align with the optimised parameters for each case. The optimum underlying SiO_2_ layer thickness was found to be approximately 60 nm for SWQE.

Since the Al nanoparticles are deposited on the front, they would exhibit both anti-reflection as well as light trapping properties^1^. While the enhanced coupling and scattering into the semiconductor layer contribute to minimised reflection in the former (SWR), the large angle scattering assists with the latter (SWQE). These two affects together aid the low SWR and high SWQE observed in these simulations. The plasmon resonance of the Al nanoparticles being in the ultra-violet region of the spectrum means that the losses due to fano resonance in the visible region normally seen for Ag and Au particles are avoided. The increased SiO_2_ thickness in both cases compete with the coupling of the scattered light from the nanoparticles into the semiconductor. In the SWR case (Fig. 4(a)) the scattering from the Al nanoparticles dominate for pitch <250 nm for thinner spacer layers where the reflectance is still lower than the thicker layers of SiO_2_. However, as the pitch increases, the particle coverage get sparse, thereby reducing the overall scattering. In this case the anti-reflection effect from the thicker SiO_2_ dominates.

Comparing the SWQE of the two sub-cells, the impact of thickness of SiO_2_ is more evident in the top cell in Fig. 5(a) because of coupling and strong scattering as mentioned earlier. Compromise between scattering due to reduced coverage and reduced coupling explains the loss in EQE for thicker spacer layers. However for the middle cell, the thickness of the spacer layer was consistently lower to the 80 nm reference in most cases as seen in Fig. 5(a). This is attributed to the difference in the optimised parameters for the top and middle cell and is evident in the J_sc_ results in Fig 3(a).

**Figure 7:**
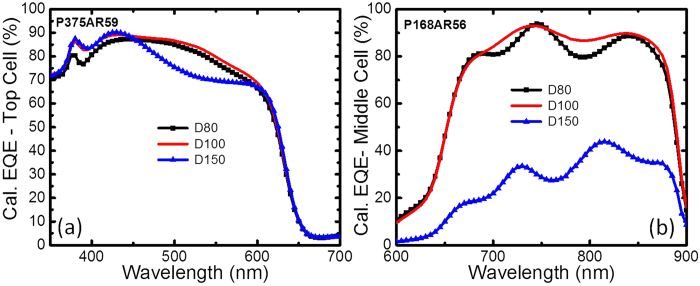
Calculated EQE spectra of the (**a**) top and (**b**) middle sub-cells configured with Al NPs of diameter 80–150 nm.

Because the earlier simulations assumed a fixed diameter of 100 nm and a height of 50 nm for the Al particles based on the reference study^11^. We fixed the height to 50 nm and varied the diameter of the particles to study the effect of size variation in the response of the sub-cells. Larger and smaller Al NPs with the same period and thickness of AR were investigated. D150H50 and D80H50 were investigated with the optimised values of P375AR59 (see Fig. 7(a)) for the top sub-cell and with P168AR56 (see Fig. 7(b)) for the middle sub-cell. Figure 7 shows that varying the diameter of the NPs did not improve the results, and on the contrary, larger particles performed worse. This is because larger particles would red-shift the resonance to longer wavelengths thus impacting the short wavelength response that the top and middle subcells would be more sensitive to. Thus, based on our calculations, D100H50 with P375AR59 and P168AR56 were regarded as the optimum configurations for the top and middle sub-cells, respectively, with a SiO_2_-based spacer layer.

#### EQE spectra of NP-incorporated 3JSC VS reference 3JSC with SLAR and DLAR

Multiple bandgap solar cells need to convert a larger spectral range to useful power with the additional challenging demand of the need for current matching. Antireflection (AR) layers are traditionally used either as a single layer or a double-layer stack optimised for the 350–900 nm range, favouring the spectral range of the current limiting top and middle sub-cells^30^. Thus, any reported enhancements must be compared to a standard cell structure. The potential for enhancements is promising only if they exceed standard cell structures. Here, we compare the performance of the Al NPs to that of standard SLAR or DLAR typically used in 3JSC.

**Figure 8:**
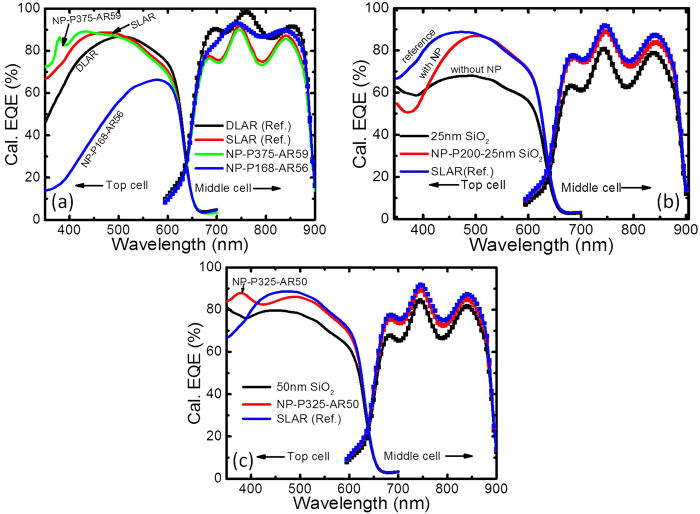
(**a**) Calculated EQE spectra of the optimum SLAR-3JSC with and without Al NPs, also including a SiO_2_-based SLAR and MgF_2_/ZnS-based DLAR reference. (**b**-**c**) Calculated EQE spectra of SLAR-3JSC with and without Al NPs for the optimised case on (**b**) 25 nm and (**c**) 50 nm SiO_2_ SLAR.

A large fraction of the scattered light from the Al NPs will be coupled into the substrate, and the exact fraction will be determined by the spacer layer thickness. It may be noted that using Al NPs on a standard thick SLAR will reduce the coupling of the scattered light into the absorber layer and thereby reduce the light absorption as discussed earlier. Because 3JSC with a SLAR of SiO_2_ yielded the overall maximum current when the thickness of SiO_2_ was 80 nm, we chose this structure as the reference for comparing the EQE spectra with Al-NP-incorporated 3JSC. Based on the optimised results, D100H50 with P375AR59 and P168AR56 were chosen for EQE to compare the performances of the top and middle sub-cells, respectively. As shown in Fig. 8(a), Al NPs with P375AR59 demonstrate higher EQE for the top cell than the SLAR reference at wavelengths below 450 nm, while Al NPs with P168AR56 show higher EQE for the middle cell at wavelengths of 600–900 nm.

For comparison, a DLAR response comprising 82 nm MgF_2_ and 44 nm ZnS^22^ is also considered. Figure 8(a) shows that the DLAR is optimised for the middle cell, although the increase in EQE is modest for the top cell compared to the SLAR. Our results show that the minimum SWR for the sub-cells with Al NPs was 7.5% (Fig. 4), which is less than that of the reference 3JSC with SLAR SiO_2_ (12.0%). The minimum SWR for NP-incorporated 3JSC appears at a configuration with a thinner underlying SLAR of 25 nm than the reference 3JSC, which agrees well with the spacer layer thickness from earlier work^11^. The maximum SWQE of 74.3% for the top cell is quite comparable to that of the SLAR reference 3JSC sample (74.2%). The maximum SWQE of 80.4% for the middle cell is slightly more than that for the SLAR reference 3JSC (i.e., 74.6%). Our results show that the pitch of the particles plays an important role in the response for the two sub-cells. However, it interesting to note that while different configurations of the NPs might have the potential to improve the sub-cells individually, the same geometry of the NPs did not yield the broadband enhancement required for the top and middle sub-cells together. Moreover, the DLAR layer was found to adequately provide broadband enhancement over the spectral region of interest for the top and middle sub-cells. We believe the Fabry-Perot oscillations for the middle cell seen in Fig. 8(b,c) arise probably due to small index differences between layers in the stack considered in the simulations. The oscillations would be smaller in practice due to thickness non-uniformities and scattering.

The effect of the spacer thickness was investigated further using thinner layers of SiO_2_ and compared to the case with and without the Al NPs. Two cases of oxide thicknesses –25 nm, corresponding to the thickness used in the reference work^11^, and 50 nm – were chosen and are shown in Fig. 8(b,c), respectively. With a 25 nm-thick SiO_2_, the Al NP-decorated 3JSC shows a better broadband performance (see Fig. 8(b)). However, the same case when compared to a 3JSC with an 80 nm-SiO_2_ SLAR did not perform as well. Similar trends were seen for the case with a 50 nm ARC in Fig. 8(c). As a further check, the performance of the GaAs photodiodes in the reference work^11^ and discussed earlier was extended to include a 100-nm SiO_2_-based SLAR for comparison. As shown in Fig. 1(d), it is clearly evident that the standard 100-nm SiO_2_ can outperform the best Al NP reported by the group. While there is an evident increase in the photocurrent for the Al NP deposited configurations compared to the flat case with the same thickness of spacer layer as with the NPs (Fig. 8(b)), it can be clearly seen that the potential for improvement is not promising compared to traditional SLAR-coated structures (reference sample). These results emphasise the importance of a proper reference comparison before the technological viability of a work is suggested. While there is scope to improve the performance of the Al NPs by a more thorough evaluation of the proper size, period, height and thickness of the underlying AR layer for a 3JSC or by using random NPs that have demonstrated broadband functionality in single-junction solar cells, it remains to be seen whether using the NPs on a thin spacer layer is sufficiently promising to outperform the antireflection coatings, which are cheaper and simpler and hence more commercially viable.

#### SWR and SWQE for embedded NP configurations

**Figure 9:**
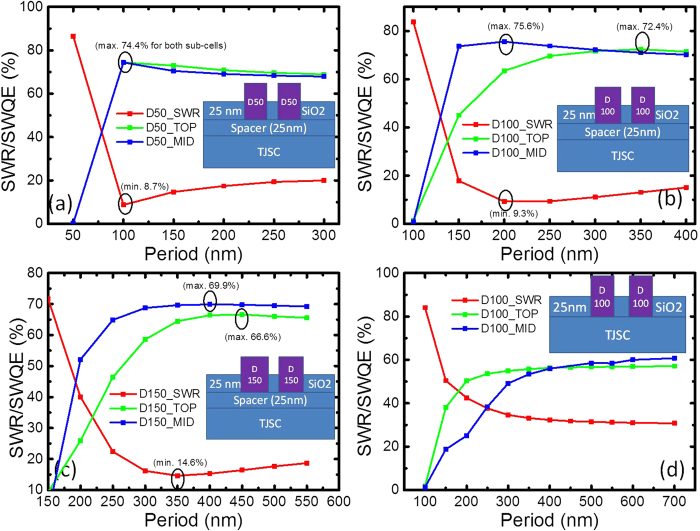
Calculated SWR and SWQE for half embedded Al NPs on top of 3JSC with (**a**–**c**) or without (**d**) a 25 nm thick SiO_2_ spacer layer for Al NPs with a diameter of (**a**) 50 nm, (**b**) 100 nm, (**c**) 150 nm, and (**d**) 100 nm.

**Figure 10:**
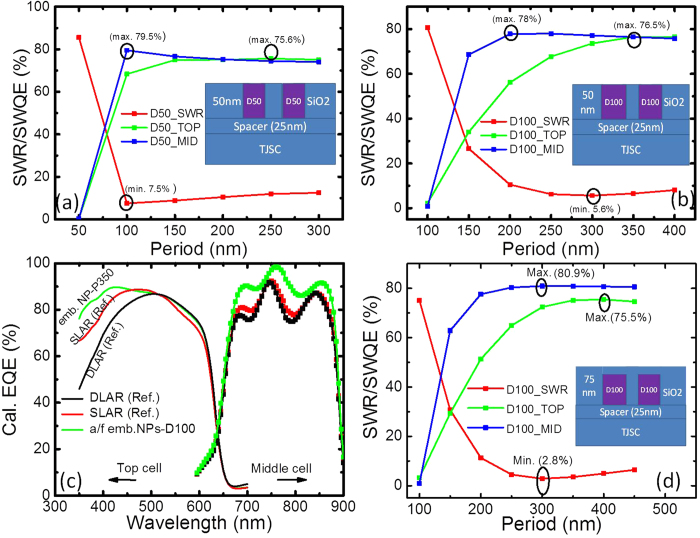
Calculated SWR and SWQE for almost fully (**a**-**c**) or fully (**d**) embedded Al NPs on top of a 25 nm thick SiO_2_ spacer layer. Al NPs with a diameter of (**a**) 50 nm, (**b**) 100 nm, (**c**) calculated EQE spectra for the best case of D100P350 and that of the SLAR and DLAR, and d) fully embedded Al NPs with a diameter of 100 nm.

Considering the fact that a thick SiO_2_ spacer/anti-reflection layer will reduce the light coupling between Al NPs and the semiconductor, we further investigate the potential of embedded Al NPs on top of a thin 25-nm spacer layer. The proximity of the NPs to the semiconductor will not compromise coupling, and the dielectric overcoating layer would provide the antireflection effect and resonance tunability of the particles. We look at SWR and SWQE for three configurations – half embedded (H), as in Fig. 9(a–d) (Fig. 9(d) was with no spacer layer), almost fully (AF) embedded, as in Fig. 10(a–c), and the fully embedded (F) case, as in Fig. 10(d). Diameters of 50, 100 and 150 nm were studied. Comparing the different sizes, for the half embedded case, the NPs with a diameter of 50 nm attained the maximum SWQE at a pitch of 100 nm for both the top and middle sub-cells, with the performance achieving that of SLAR. The NPs with a diameter of 100 nm yielded different optimum configurations for the top and middle cells. The 150-nm diameter case did not perform as well as the other two cases, as shown in Fig. 9(c).

The case of NPs directly on top of the window layer without the 25-nm SiO_2_ spacer was simulated as well (Fig. 9(d) for the 100 nm diameter NP). Comparing the case with and without the spacer layer, there was a clear benefit to having a thin spacer layer due to an enhanced driving field, which has been previously reported by our group^7^. For the almost-fully embedded case, the SWR drops and SWQE increase compared to the half embedded case. This result is attributed to the good antireflection properties of the combined SiO_2_ and Al NP case. It is interesting to note that for the case of almost-fully embedded NPs with a diameter of 100 nm and a pitch of 350 nm, SWQE for the top and middle cells are nearly identical, with a value of 76.5%, which is higher than the SLAR reference (74.2% and 74.6% SWQE for top and middle cells, respectively), as shown in Fig. 10(c). The improved performance of the cells compared to the non-embedded case here is because of the less back-scattered light lost from the nanoparticles into air, signifying less reflectance. The reduction in reflection for the D100H50 nanoparticles with the same 25 nm SiO_2_ layer at P350nm in Figs 9(a),10(b) and 10(d) for varying degrees of embedding is seen. While a larger fraction of the light will be scattered into the SiO_2_ spacer layer (owing to the larger refractive index compared to air) for the embedded case, there is a greater potential for it to be coupled back into the top cell as the layer is non-absorbing and has a lower escape cone. This effect is clearly evident in the case for the fully embedded case where the outcoupling losses into air are significantly reduced. This explains the better response for the fully embedded case compared to the SLAR, although the response from the sub-cells were different. A slight shift in the resonance position is also expected due to the change in the dielectric environment which may vary the scattering properties. Another reason is the better coupling of the scattered light into the cell. Embedding the nanoparticles allows thinner spacer layers (25 nm as compared to ~55 nm) allowing better interaction of light with the semiconductor layer. Table 1 summarises the SWR and SWQE results of all the investigated structures for a 3JSC, including the case with SLAR and DLAR.

Our results show that while the nanoparticles have the potential for improving the performance of a 3JSC, achieving broadband light trapping with nanoparticles is challenging due to performance mismatch between the sub cells. We note that in this work, we have focused on particles sizes close to a 100 nm diameter with fixed height and shape, but there exists a huge parameter space for optimisation, which might possibly yield a suitable broadband scattering layer in Al NPs suitable for 3JSC including the use of randomly distributed nanoparticles which was not considered in the simulations. With careful design and optimisation, random subwavelength nanostructures have the potential to provide broadband light trapping^31^, however they need to be reproducible and adapt to the current matching conditions of 3JSC cells which can limit the performance of cells. One of the important conclusions from this work is that, new light trapping techniques need to be able to compete with existing commercial technologies to be viable. However, the cost factor for fabricating embedded structures on commercial cells should be carefully considered.

## Conclusions

We have discussed the potential of periodic Al NPs for improving the EQE of GaInP/GaInAs/Ge triple junction solar cells. Parameters including the period of the NPs and the thickness of the underlying spacer layer were combined to optimise the performance of the top and middle sub-cells. Our results show that the optimum configurations of NPs on 3JSC for the top and middle sub-cells are not identical. The calculated EQE for the top sub-cell of 3JSC with optimum NPs is higher than the reference 3JSC with a SiO_2_-based single antireflection layer for wavelengths less than 500 nm, while 3JSC with optimum NPs demonstrate an increase in EQE for the middle sub-cell at wavelengths of 690–850 nm. Our results highlight the importance of comparing the performances of devices to standard references, and it can be concluded that though Al NPs placed directly on a spacer layer can improve the performance of 3JSC in certain parts of the spectrum, they cannot surpass the performance of a good antireflection layer. However, for configurations of NPs embedded in SiO_2_, it was found that there is potential for broadband improvement compared to standard antireflection coatings with the added advantage of obtaining similar responses from the top and middle cells, but subject to cost considerations.

## Additional Information

**How to cite this article**: Yang, L. *et al.* Can plasmonic Al nanoparticles improve absorption in triple junction solar cells? *Sci. Rep.* **5**, 11852; doi: 10.1038/srep11852 (2015).

## Figures and Tables

**Table 1:** SWR and SWQE values of all the investigated configurations used in this study.

Configuration		SWR(%)	SWQE-top(%)	SWQE-mid.(%)	Note
SLAR-reference		**13.8**	**74.2**	**74.6**	(80 nm SiO_2_)
DLAR-reference		3.1	73.2	83.3	
NPs-on-3JSC	D100-AR59-P375	11.5	74.3	73.2	Opt. for top
	D100-AR56-P168	14.5	49.5	80.4	Opt. for middle
Embedded NPs-on-3JSC (with 25 nm spacer layer)	**H-D50**	**8.7 @P100**	**74.4@P100**	**74.4@P100**	Half embedded
	H-D50	7.5@P100	75.6@P250	79.5@P100	
	AF-D100	5.6@P300	76.5@P350	78@P250	Almost fully embedded
	*AF-D100-P350	**6.53**	**76.5**	**76.4**	
	F-D100	2.8@P300	75.5@P400	80.9@P300	Fully embedded
	**F-D100-P400**	**4.9**	**75.5**	**80.7**	

Here, *H* stands for half embedded, *AF* for almost fully embedded and *F* for the completely embedded case.* Shows the best case where the performances of the top and middle cells in the embedded-NP-modified 3JSC are simultaneously better than those in the SLAR reference 3JSC and yield similar values.

## References

[b1] Pillai S. *et al.* Surface plasmon enhanced silicon solar cells. J. Appl. Phys. 101, 093105–093105 (2007).

[b2] Atwater H. A. & Polman A. Plasmonics for improved photovoltaic devices. Nat. Mater. 9, 205–213 (2010). 20168344 10.1038/nmat2629

[b3] Green M. A. & Pillai S. Harnessing plasmonics for solar cells. Nat. Photon. 6, 130–132 (2012).

[b4] Spinelli P. *et al.* Plasmonic light trapping in thin-film Si solar cells. J. Opt. 14, 024002 (2012).

[b5] Nakayama K., Tanabe K. & Atwater H. A. Plasmonic Nanoparticle Enhanced Light Absorption in GaAs Solar Cells. Appl. Phys. Lett. 93, 121904 (2008).

[b6] Grandidier J. *et al.* Solar cell efficiency enhancement via light trapping in printable resonant dielectric nanosphere arrays. Phys. Status Solidi A. 210, 255–260 (2013).

[b7] Pillai S., Beck F., Catchpole K. R., Ouyang Z. & Green M. A. The effect of dielectric spacer thickness on surface plasmon enhanced solar cells for front and rear side depositions. J. Appl. Phys. 109, 073105 (2011).

[b8] Yang Y. *et al.* Enhanced light trapping for high efficiency crystalline solar cells by the application of rear surface plasmons. Sol. Energ. Mat. Sol Cells. 101, 217–226 (2012).

[b9] Zhang Y. *et al.* Improved multicrystalline Si solar cells by light trapping from Al nanoparticle enhanced antireflection coating. Opt. Mat. Exp. 3, 489–495 (2013).

[b10] Akimov Y. A. & Koh W. S. Resonant and non-resonant plasmonic nanoparticle enhancement for thin-film silicon solar cells. Nanotechnology 21, 235201 (2010). 20463389 10.1088/0957-4484/21/23/235201

[b11] Hylton N. P. *et al.* Loss mitigation in plasmonic solar cells: Aluminium nanoparticles for broadband photocurrent enhancements in GaAs photodiodes. Sci. Rep. 3, 2874 (2013). 24096686 10.1038/srep02874 PMC3791440

[b12] Temple T. L. & Bagnall D. M. Optical properties of gold and Aluminium nanoparticles for silicon solar cell applications. J. Appl. Phys. 109, 084343 (2011).

[b13] Chen X., Jia B. *et al.* “Exceeding the limit of plasmonic light trapping in textured screen-printed solar cells using Al nanoparticles and wrinkle-like graphene sheets,” Light Sci. Appl. 2, e92 (2013).

[b14] Massa E. *et al.* Diffractive interference design using front and rear surface metal and dielectric nanoparticle arrays for photocurrent enhancement in thin crystalline silicon solar cells. ACS Photonics 1, 871–877 (2014).

[b15] Cotal H. *et al.* III–V multijunction solar cells for concentrating photovoltaics. Energy Environ. Sci. 2, 174–192 (2009).

[b16] King R. R. *et al.* 40% efficient metamorphic GaInP GaInAs Ge multijunction solar cells. Appl. Phys. Lett. 90, 183516 (2007).

[b17] Geisz J. F. *et al.* 40.8% efficient inverted triple-junction solar cell with two independently metamorphic junctions. Appl. Phys. Lett. 93, 123505 (2008).

[b18] Philipps S. P. *et al.* Present Status in the Development of III–V Multi-Junction Solar Cells. Next Generation of Photovoltaics. Next Generation of Photovoltaics, Springer Series in Optical Sciences 165. (Springer-Verlag: Heidelberg, 2012).

[b19] King R. R. *et al.* Band-gap-engineered architectures for high-efficiency multijunction concentrator solar cells. 24th European Photovoltaic Solar Energy Conference and Exhibition, Hamburg, Germany, 2009.

[b20] Kurtz S. Opportunities and Challenges for Development of a Mature Concentrating Photovoltaic Power Industry. www.nrel.gov/docs/fy13osti/43208.pdf. (Accessed: 17th October 2013)

[b21] Song Y. M., Jeong Y., Yeo C. & Lee Y. T. Enhanced power generation in concentrated photovoltaic using broadband antireflective cover glasses with moth eye structures. Opt. Express 20, A916 (2012). 23326839

[b22] Homier R. *et al.* Antireflection Coating Design for Triple-Junction III–V/Ge High-Efficiency Solar Cells Using Low Absorption PECVD Silicon Nitride. IEEE J. Photov. 2, 393–397 (2012).

[b23] Sai H., Kanamori Y., Arafune K., Ohshita Y. & Masafumi Y. Light Trapping Effect of Submicron Surface Textures in Crystalline Si Solar Cells. Prog. Photovoltaics. 15, 415–423 (2007).

[b24] Yamaguchi M., Tatsuya T., Kenji A. & Nicholas E.-D. Multi-junction III–V solar cells: current status and future potential. Sol. Energy 79, 78–85 (2005).

[b25] Jones R. K., Ermer J. H., Fetzer C. M. & King R. R. Evolution of Multijunction Solar Cell Technology for Concentrating Photovoltaics. Jpn. J. Appl. Phys. 51, 10ND01 (2012).

[b26] McConnell R. D. & Martha S.-D. Multi junction Photovoltaic Technologies for High Performance Concentrators. http://www.nrel.gov/docs/fy06osti/39866.pdf. (Accessed: 20th October 2013)

[b27] Baur C. *et al.* Triple-Junction III–V Based Concentrator Solar Cells: Perspectives and Challenges. J. Sol. Energy Eng. 129, 258–265 (2007).

[b28] Nishioka K. *et al.* Evaluation of InGaP/InGaAs/Ge triple-junction solar cell and optimization of solar cell’s structure focusing on series resistance for high-efficiency concentrator photovoltaic system. Sol. Energ. Mat. Sol Cells. 90. 1308–1321 (2006).

[b29] Palik E. D. Handbook of Optical Constants of Solids. (Academic Press: Orlando, 1985).

[b30] Aiken D. J. High performance anti-reflection coatings for broadband multi-junction solar cells. Sol. Energ. Mat. Sol Cells . 64, 393–404 (2000).

[b31] Yu Z., Raman A. & Fan S. Fundamental limit of nanophotonic light trapping in solar cells. Proc. Natl. Acad. Sci. U.S.A, 107, 17491–17496 (2010). 20876131 10.1073/pnas.1008296107 PMC2955111

